# Space-time analysis of pneumonia hospitalisations in the Netherlands

**DOI:** 10.1371/journal.pone.0180797

**Published:** 2017-07-13

**Authors:** Elisa Benincà, Michiel van Boven, Thomas Hagenaars, Wim van der Hoek

**Affiliations:** 1 Centre for Infectious Disease Control, National Institute for Public Health and the Environment, Bilthoven, The Netherlands; 2 Department of Bacteriology and Epidemiology, Wageningen Bioveterinary Research, Lelystad, The Netherlands; Columbia University, UNITED STATES

## Abstract

Community acquired pneumonia is a major global public health problem. In the Netherlands there are 40,000–50,000 hospital admissions for pneumonia per year. In the large majority of these hospital admissions the etiologic agent is not determined and a real-time surveillance system is lacking. Localised and temporal increases in hospital admissions for pneumonia are therefore only detected retrospectively and the etiologic agents remain unknown. Here, we perform spatio-temporal analyses of pneumonia hospital admission data in the Netherlands. To this end, we scanned for spatial clusters on yearly and seasonal basis, and applied wavelet cluster analysis on the time series of five main regions. The pneumonia hospital admissions show strong clustering in space and time superimposed on a regular yearly cycle with high incidence in winter and low incidence in summer. Cluster analysis reveals a heterogeneous pattern, with most significant clusters occurring in the western, highly urbanised, and in the eastern, intensively farmed, part of the Netherlands. Quantitatively, the relative risk (RR) of the significant clusters for the age-standardised incidence varies from a minimum of 1.2 to a maximum of 2.2. We discuss possible underlying causes for the patterns observed, such as variations in air pollution.

## Introduction

Community acquired pneumonia (CAP) is one of the major causes of hospitalisation and death in developing as well as in developed countries. Alarmingly, the burden of pneumonia has been increasing [1, 2] and according to a recent study in England, this trend cannot be explained just by changes in demography, admission practices or diagnosis codes [2].

Like in other countries, in the Netherlands, CAP is a major health problem with 40,000–50,000 hospital admissions per year. In the large majority of hospital admissions for pneumonia the etiologic agent is not determined and the common ICD-10 discharge diagnosis is 'pneumonia, organism not specified'. The typical seasonal pattern in temperate climatic regions with sharp increases during the winter is generally attributed to complications of influenza virus infections, especially co-infection with bacteria such as *Streptococcus pneumoniae* among the elderly (>65 years old). However, there are several reasons for obtaining more insight in variation in time and space, epidemiological determinants, and causative agents of severe pneumonia. First, a previous study using hospital discharge data, retrospectively showed clusters of pneumonia hospitalisations that were not recognised at the time and that were likely caused by Q-fever, two years before a large Q-fever epidemic in the Netherlands was detected [3]. As a result, there was a delay in implementation of control measures. Second, triggered by the Q-fever epidemic, there are concerns among professionals and the public about possible health risks associated with intensive animal husbandry. Studies in the livestock-dense south of the Netherlands show an increased incidence of pneumonia in people living close to poultry farms, for which there is no definite explanation yet [4]. Third, a recent study in the Netherlands showed that CAP is often caused by atypical (zoonotic and non-zoonotic) pathogens, especially outside the influenza season and among male patients (< 60 years old) [5].

Although studies have been carried out to understand what factors drive the onset of pneumonia, only a handful examine the spatio-temporal dynamics of pneumonia [3, 6, 7]. Here, we investigate the spatio-temporal patterns of pneumonia hospital admission data at the national level to identify potential high-risk areas.

## Material and methods

No consent was given because the data were analyzed anonymously.

### Data

**Fig 1:**
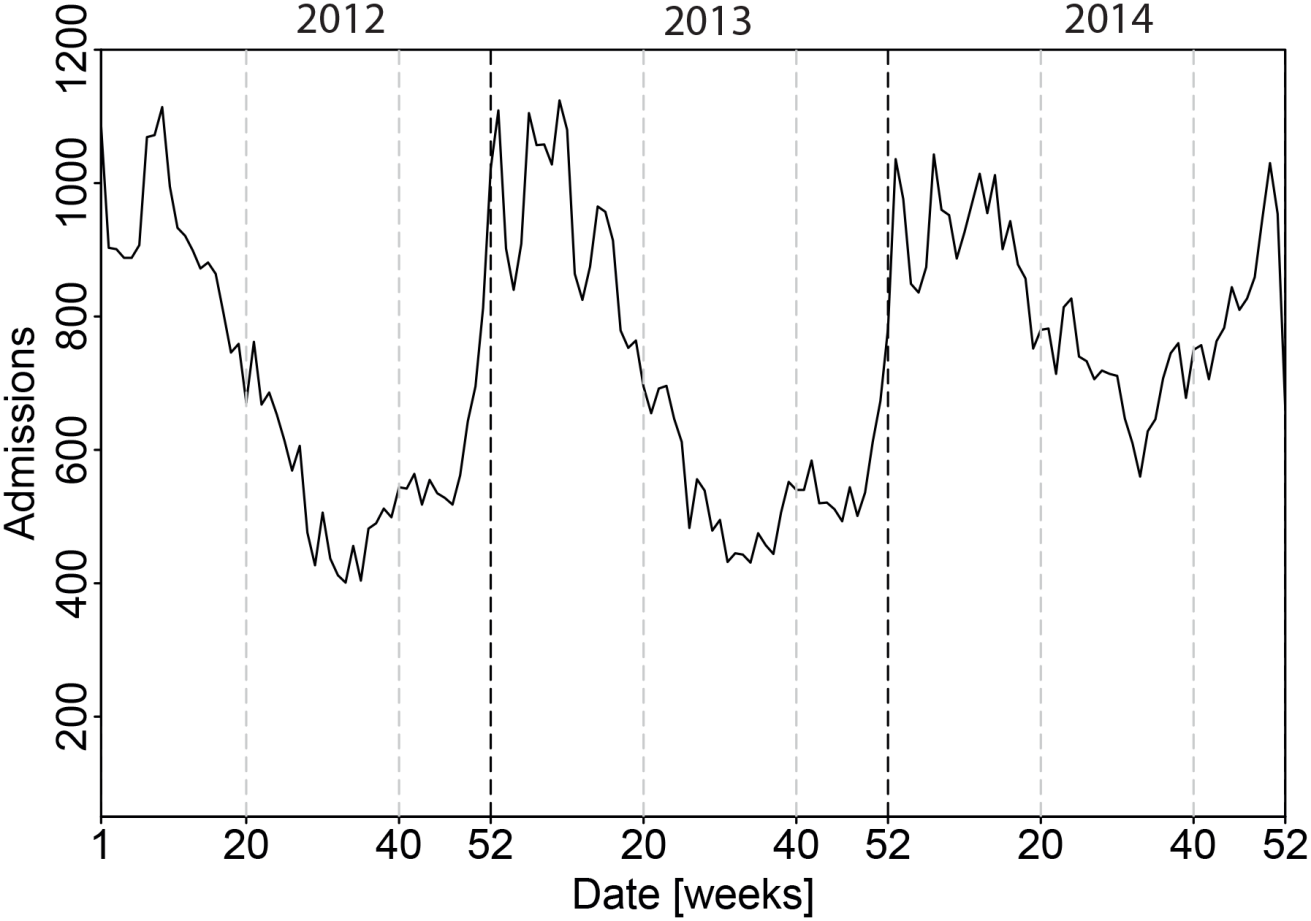
Time series of unspecified pneumonia (ICD-10 discharge diagnosis J18) hospitalisation cases plotted on a weekly basis.

We analysed hospital admission data for the years 2012, 2013, and 2014 obtained from Dutch Hospital Data (DHD). DHD is a national register collecting medical diagnoses of people that have been hospitalised in a Dutch hospital or medical center. DHD had a registration coverage of about 80% in 2012 and 2013, which increased to approximately 90% in 2014. The database is anonymised and for each hospital admission contains a patient ID number, an ID number of the hospital, the date of birth, the date of admission and dismissal, and the main and secondary discharge diagnoses. The main and secondary discharge diagnoses are coded according to the International Classification for Diseases (ICD-10). If a patient was hospitalised several times in the same year with the same diagnosis, this patient was counted only once. The data are available at four digits (out of six) postal code level, which is the highest level of spatial resolution available within the constraints set by Dutch privacy legislation. We are mainly interested in the cases of pneumonia with unknown etiology, which accounts for more than 86% of the total admissions (see Table 1). Therefore, throughout we included only cases coded as “Pneumonia organism unspecified”. This yielded 115,036 records. Weekly time series of hospital admissions for unspecified pneumonia are shown in Fig 1. The data analysed here include patients of all ages (see S1 Table, for an overview of the number of cases per each age group).

**Table 1:** Counts of hospital admissions for pneumonia in 2012, 2013 and 2014 for different ICD-10 discharge codes. In the majority of cases the etiological agent is not determined and the ICD discharge diagnosis is ‘pneumonia organism unspecified’. The proportion of pneumonia cases in 2014 is significantly higher than in 2012 and in 2013 (One sided Pearson’s Chi square statistics, p-value<0.001).

Etiological agent ICD-10 code	Pneumonia organism specified J13, J14, J15, J16	Percentage of the total (%)	Pneumonia organism unspecified J18	Percentage of the total (%)	Total	Population	Proportion of total population hospitalized for pneumonia
Year							
**2012**	5618	13	36604	87	42222	16727375	0.0025
**2013**	5945	14	36127	86	42072	16777010	0.0025
**2014**	6837	14	42305	86	49142	16826675	0.0029

### Incidence and age adjusted incidence

We built yearly incidence maps at different levels of spatial resolution, and here we present a map at the municipality level. We note that elderly people are more prone to develop pneumonia and that the age distribution is not homogeneous across the country. As our aim is to assess whether some areas are at higher risk of pneumonia independently of the local age distribution, we also calculated the age adjusted incidence (*AAI*) for each municipality region by using the direct standardisation method [8] as follows:

$$ASI_{ij}=\frac{n_{ij}}{pop_{ij}}$$ (1.1)

$$ADSP_{i}=\frac{popstand_{i}}{\sum popstand_{i}}$$ (1.2)

$$AAI_{j}=\sum \left(ASI_{ij}*ADSP_{i}\right)$$ (1.3)

First, for each year we chose the Dutch population of that year as the standard population and we divided it in four age classes: [0–25); [25–45); [45–65) and 65+. Second, for each age class *i* we calculated the age specific incidence (*ASI*) of pneumonia at municipality level with *n*_*i j*_ and *pop*_*i j*_ being the number of pneumonia cases and the total population for a specific age class *i* within a municipality *j* (Eq 1.1). Then we calculated the age distribution of the standard population (*ADSP*) with *popstand*_i_ being the standard population in each age class *i*. (Eq 1.2). Finally, we calculated the age adjusted incidence (*AAI*) for each municipality *j* (Eq 1.3).

To obtain an overview of the seasonal behavior of the spatial distribution of pneumonia incidence, we also calculated for each season of the year the age-adjusted incidence averaged over the three years. For the seasonal incidence we used as standard the Dutch population of 2014.

In order to build the municipality incidence maps, we lumped the postal codes at the municipality level. The number of municipalities changed over the years. In 2012, the Netherlands counted 415 municipalities that were reduced to 408 in 2013 and to 403 in 2014. For each year, we used the partition of that year. For the seasonal incidence, we used the partition of 2014.

All incidence maps have been built using the R package Maptools for reading geographic data (https://www.r-project.org).

### Spatial scan statistics

In order to detect spatial clusters of high rates of pneumonia cases we applied the SaTScan spatial scan statistics [9, 10] using the SaTScan software (SaTScan version 9.4, http://www.satscan.org/).

The spatial scan statistic centers a circular window of flexible radius in the centroid of the four-digit postal code areas, and compares observed and expected numbers of cases inside and outside the window. The expected cases are calculated assuming that the number of cases in each area is Poisson distributed. Because we are interested in localised clusters of high rates within the circle rather than areas with low rates outside the circle, we impose an upper limit on the radius of the scanning window equal to 10% of the population at risk.

As for the incidence, we aimed to detect spatial clusters of pneumonia independently of the spatial distribution of age classes. For this purpose, we applied the SaTScan statistic by adjusting for the covariate age class in the analysis.

The four-digit postal code was not always available for each patient. Therefore, for the incidence maps and the SaTScan analysis we selected only the data provided with postal codes. This yielded 36,443 cases for 2012, 35,995 cases for 2013 and 42, 267 cases for 2014.

### Wavelet analysis

Although with the SaTScan software it is also possible to detect temporal and spatio-temporal clusters, the method is in principle not suited for time series that are non-stationary as is the case for many epidemiological and ecological data [11, 12]. The data of pneumonia are clearly non-stationary (Fig 1).

For the (spatio) temporal analysis we therefore used wavelet analysis instead, a technique particularly suited for non-stationary time series. In order to compare the temporal patterns of pneumonia at spatial scales we divided the Netherlands in five regions. We lumped the age corrected incidence of pneumonia to obtain a time series for each of the five regions. We then performed wavelet analysis to each of the time series.

Wavelet analysis makes use of a local periodic function (the wavelet) to decompose fluctuations of time series observed during a small stretch of time into a series of different periodicities. The relative importance of periodicities (wavelet power) is then plotted in contour plots as a function of time. In this way, both the periodicity and the timing of the fluctuations can be identified. More detailed information on wavelet analysis can be found in [11–16] and in S1 Appendix.

### Wavelet cluster analysis

With the wavelet spectra of the different regions at hand, we quantify the similarity between the patterns using a Maximum Covariance Analysis (MCA), yielding a dissimilarity matrix of the wavelets [17, 18]. Subsequently, the dissimilarity matrix is used to construct a cluster tree based on the WARD agglomeration criterion. Further information on wavelet clustering and its application in ecological and epidemiological studies can be found in [17–20], and in S1 Appendix.

Wavelet spectra and the wavelet cluster tree have been computed using the R package ‘biwavelet’ by Gouhier, Grinsted and Simko (http://biwavelet.r-forge.r-project.org/).

## Results

The temporal dynamics of pneumonia hospitalisations display a strong seasonal pattern with peaks in winter and troughs in summer (Fig 1). This seasonal oscillatory behavior is observed in all years, although the difference in number of cases between winter and summer is less pronounced in 2014. In addition, there is a substantial increase in the total number of cases in 2014 compared with the previous years (see also Table 1).

**Fig 2:**
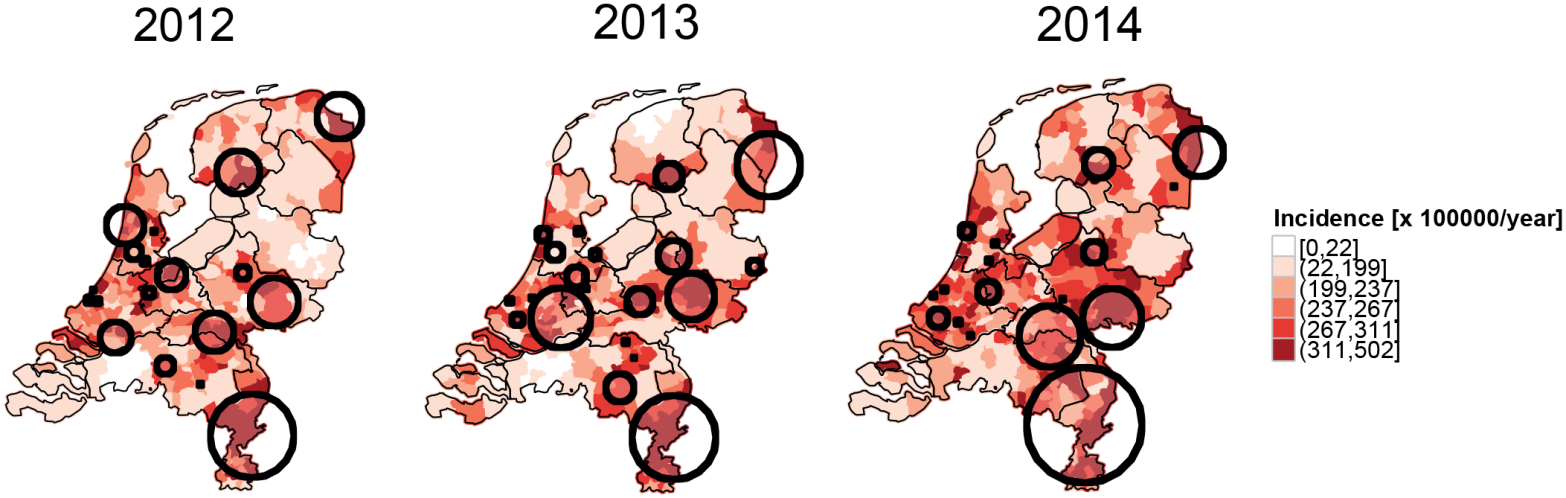
Maps of incidence of unspecified pneumonia cases at municipality level for the years 2012, 2013 and 2014. Black circles represent significant clusters (p<0.05) identified whilst imposing a 10% upper limit and choosing a non-overlapping criterion. The incidence intervals in the colorbar represent the quantiles of the pneumonia incidence in 2014.

The incidence of pneumonia is heterogeneously distributed across the country (Fig 2). A zone of high incidence stretches from west to east to south-east (redder colors in the maps in Fig 2a–2c). This pattern is confirmed by SaTScan spatial cluster analysis. The majority of significant clusters (black circles in Fig 2) occur in the western, in the eastern and in the south eastern part of the Netherlands. This spatial pattern is robust and highly consistent across the years (Fig 2a–2c). Quantitatively, the relative risk (RR) of the significant clusters varies from a minimum of 1.15 to a maximum of 8.6.

**Fig 3:**
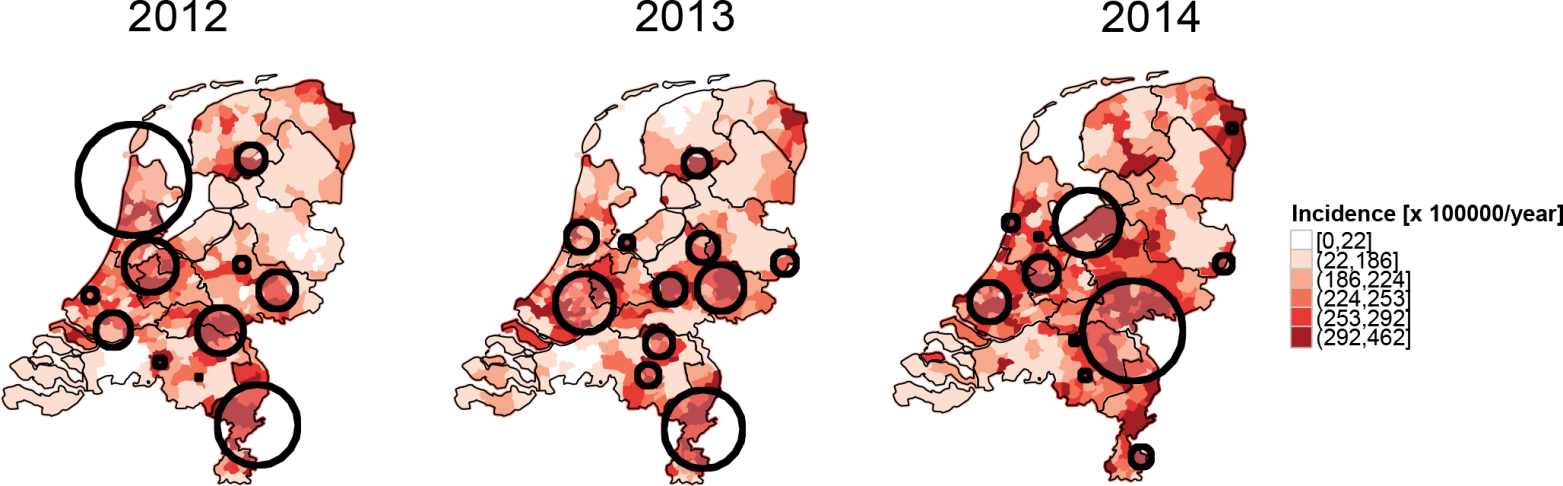
Maps of age adjusted incidence of unspecified pneumonia cases at municipality level for the years 2012, 2013 and 2014. Black circles represent significant clusters (p<0.05) identified whilst imposing a 10% upper limit and choosing a non-overlapping criterion. The clusters in Sat Scan have been adjusted by taking age classes as covariate in the analysis. The incidence intervals in the colorbar represent the quantiles of the pneumonia incidence in 2014.

Elderly people are at higher risk of developing pneumonia, and the age classes are not equally distributed within the country. So one might argue that the observed spatial patterns result from differences in age distributions. Hence, to assess whether some areas are at higher risk of pneumonia independently of the age class distribution, we corrected the incidence and the SaTScan analysis by taking age into account (see Methods). Again, the spatial pattern is robust (Fig 3a–3c): high age-corrected incidence show a spatial distribution similar to the high age uncorrected incidence (Fig 2a–2c). Furthermore, there are fewer localised clusters than in the crude, unadjusted analysis. Interestingly, the clusters occur in an area with heterogeneous characteristics. The west of the Netherlands is a densely populated area (indicated by darker colors in S2 Fig) with low farm activity (indicated by lighter colors in S2 Fig). In contrast, the east and the south east of the Netherlands are characterised by lower population density (indicated by lighter colors in S2 Fig) and high farm activity (indicated by darker colors in S2 Fig). Quantitatively, the relative risk (RR) of the significant clusters vary from a minimum of 1.2 to a maximum of 2.2.

**Fig 4:**
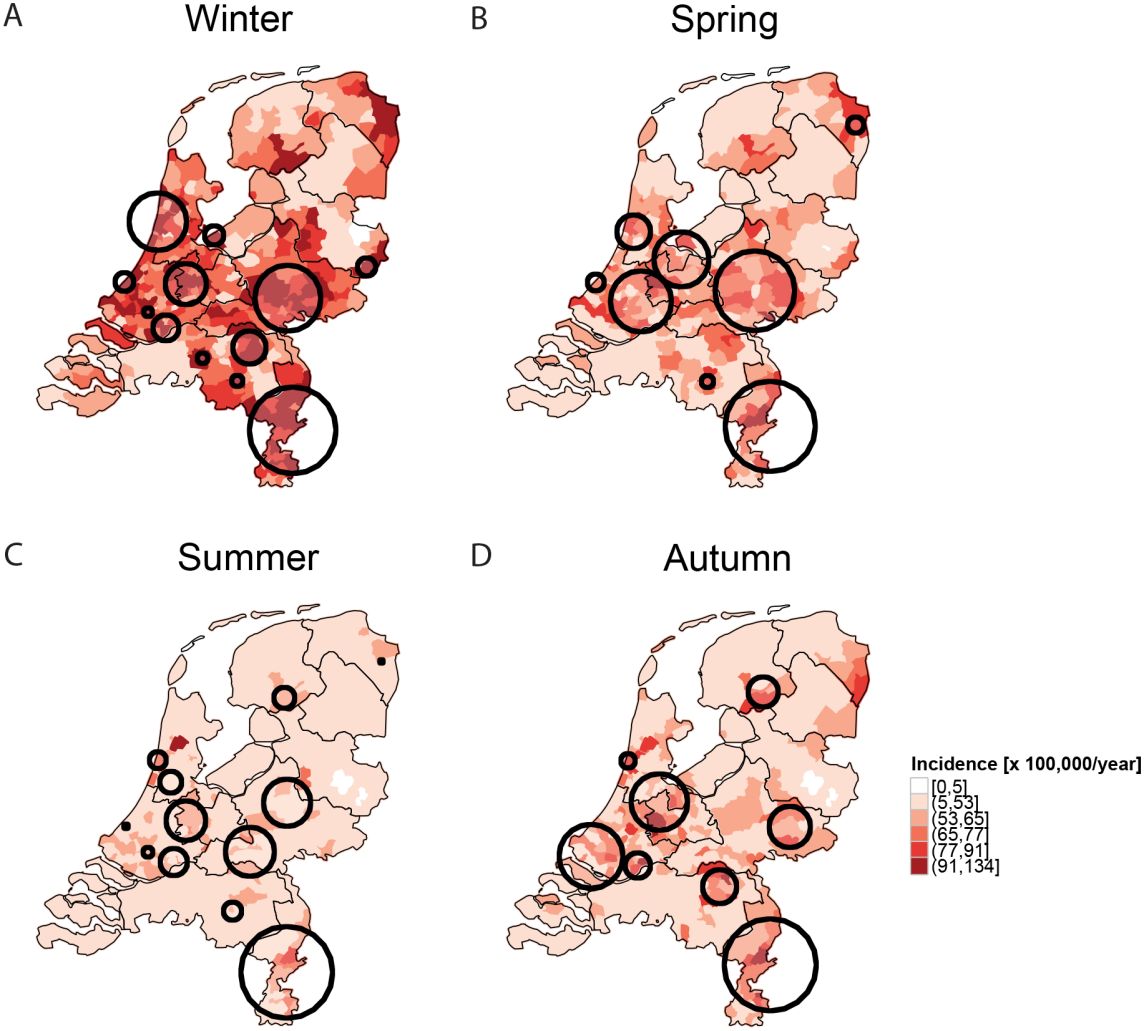
Maps of seasonal age-adjusted incidence of unspecified pneumonia cases at municipality level. Black circles represent significant clusters (p<0.05) identified whilst imposing a 10% upper limit. The clusters are calculated by taking all the seasonal cases of the three years and the incidence is calculated by averaging over three years. The clusters in SaTScan have been adjusted by taking age class as covariate in the analysis.

For each season of the year, we calculated age-adjusted incidence averaged over the 3 years, and performed an age-adjusted SaTScan spatial analysis (Fig 4a–4d). A clear seasonal pattern characterizes the age corrected incidence maps of pneumonia: higher incidence occurs mainly in winter, followed by spring, autumn, and summer. Despite the large difference in incidence between the four seasons, the spatial pattern remains robust among/across the four maps (Fig 4a–4d). In fact, as for the yearly incidence the significant seasonal spatial clusters are in an area that extends from west to east and south east of the Netherlands (Fig 4a–4d). Quantitatively, the relative risk (RR) of the significant clusters vary from a minimum of 1.2 to a maximum of 3.4. This spatial pattern is robust even when we focus on the seasonal age-adjusted clusters of each year (S1 Fig).

In addition, differences between low-versus high-incidence regions are consistently more than one order of magnitude. In fact, in the yearly maps (Fig 3), the incidence in the dark red areas ranges from 292 to 462 per 100,000 per year, while the incidence in the white areas ranges from 0 to 22 per 100,000 per year. In the seasonal maps (Fig 4), the contrast between high and low incidence areas is also remarkable. Even in summer, when incidence is much lower than in the other seasons, low and high incidence values differ by more than one order of magnitude: some municipalities (white areas) are characterised by an average incidence of 0–5 per 100,000 per year, and others (dark red areas) by an average incidence 91–134 per 100,000 per year.

**Fig 5:**
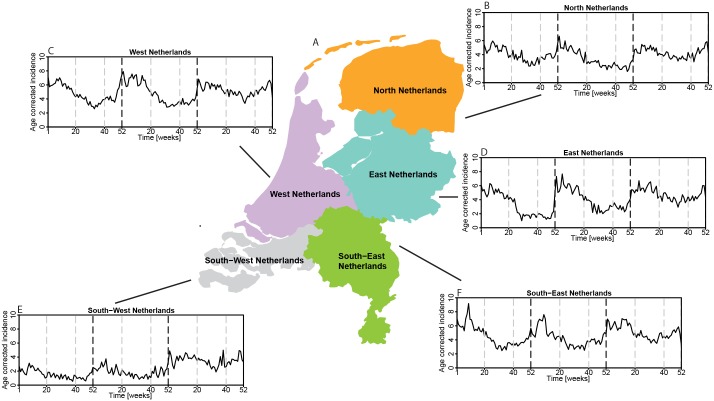
Map of the Netherlands divided in five regions. Time series of age-adjusted incidence [x 100,000 per week] of unspecified pneumonia for the five regions of the Netherlands (b-f).

We were also interested in capturing differences in the temporal dynamics of pneumonia at the spatial scale. Because of the non-stationarity of the data, we could not perform a spatio-temporal SaTScan analysis. Instead, we applied wavelet cluster analysis (Methods) to the time series of age-adjusted pneumonia of five regions in the Netherlands (Fig 5a). The temporal dynamics of pneumonia is characterised by higher values in winter and lower values in summer in all five regions (Fig 5b–5f). However the seasonal differences are stronger in West Netherlands (in purple on the map), in East Netherlands (light blue) and in South East (green) than in the other two regions.

**Fig 6:**
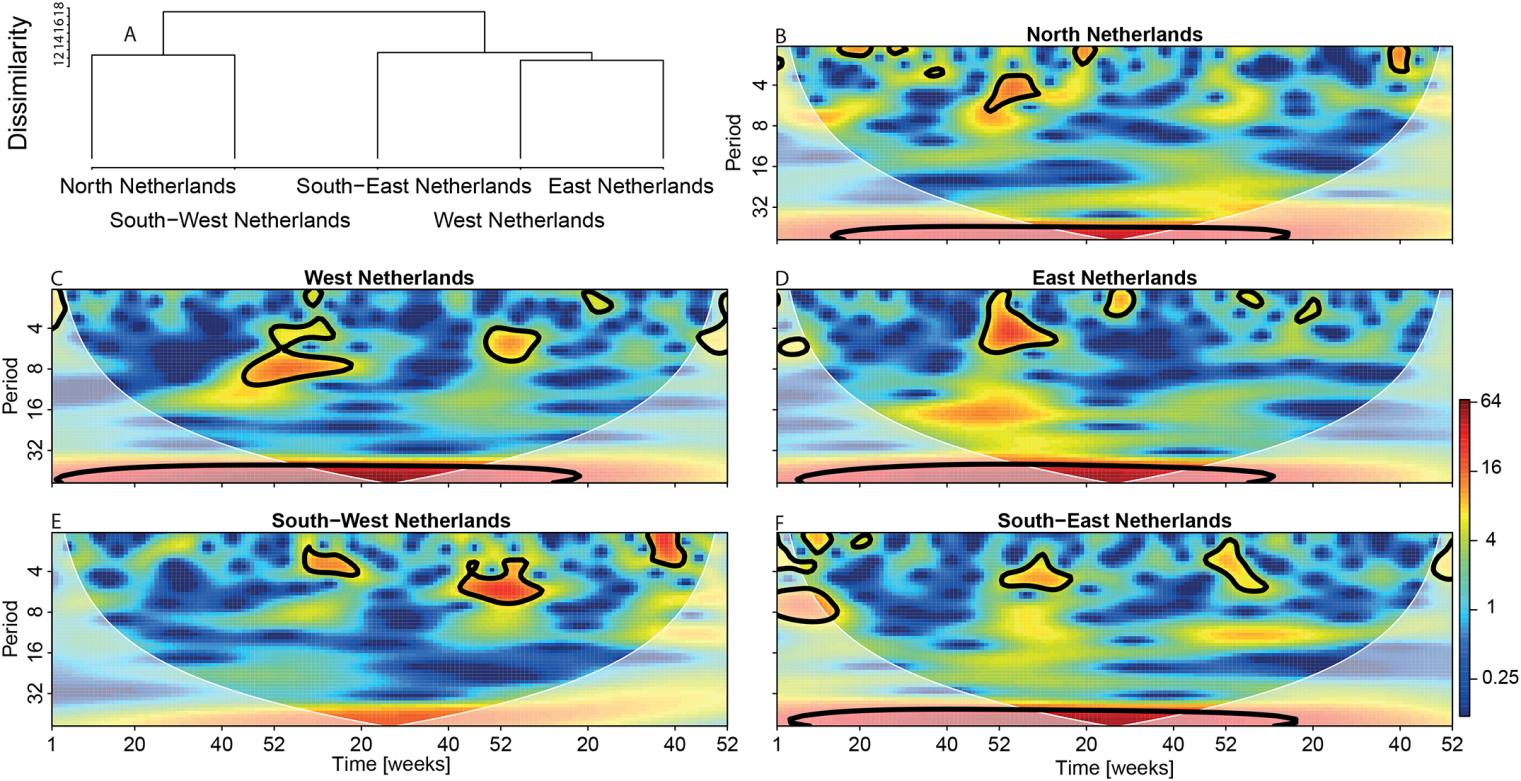
Wavelet power spectra of the age adjusted incidence for the five regions of the Netherlands. Color codes represent wavelet power and areas inside the black contour lines correspond to 95% confidence regions where the power is higher than the power of red noise with the same autocorrelation coefficient as the data. Transparent areas on the left and right hand sides of the plots represent the cone of influence, which is a region where edge effects are important. A cluster tree has been constructed (a) based on the dissimilarity matrix of the wavelets power spectra.

This result is confirmed by wavelet analysis. Wavelet spectra revealed a significant dominant periodicity of about 52 weeks (red areas inside the black lines) in all regions except for South-West Netherlands (Fig 6b–6d). In the time lag between week 52 of 2012 and week 13 of 2013 wavelet analysis identifies a significant periodicity of about 4–8 weeks for all regions but South-West Netherlands. This coincides with the peaks of pneumonia cases occurring at the beginning of 2013 (see Figs 1, 5b–5d and 5f). We also built a tree based on a wavelet dissimilarity matrix (Fig 6a). The dissimilarity matrix identifies two levels of aggregation: at one side West Netherlands and North Netherlands and at the other side South-East, West and East Netherlands. If we base the analysis on normalised square-root transformed data (to homogenize the variance), the clustering remains comparable, the only difference being that the South-West and the North Netherlands don’t cluster together anymore (S3 Fig). Strikingly, the partition detected by the cluster analysis coincides with the partition of high and low incidence areas detected by the SatScan analysis.

## Discussion

Here, we provide a comprehensive analysis of space-time clustering of pneumonia hospitalisations by using two different statistical approaches. Our results show that pneumonia hospital admissions are strongly clustered both in space and in time. The spatial distribution of pneumonia is heterogeneous, and differences between high and low incidence areas can be more than an order of magnitude.

So far not many studies have described the spatial patterns of pneumonia. This is, to the best of our knowledge, the first time, that such a huge variation in incidence is observed at such a small spatial scale. A previous study of pneumonia hospitalisation in the province of Ontario in Canada also shows remarkable differences in the pneumonia rates between the different counties [6]. However, the surface area of Ontario is more than an order of magnitude larger/bigger than the surface of the Netherlands, and strong geographic and climatic differences exist among the Ontario counties. The Netherlands in contrast, is both geographically and climatologically more homogeneous. However, levels of urbanisation and livestock activity differ across the country (S2 Fig), and this might explain some of the heterogeneities.

The dissimilarity matrix based on the wavelet spectra reveals two aggregation levels. However, this classification results from a difference in incidence rather than from a difference in temporal dynamics. In fact, the dissimilarities between the different regions (Fig 6a, S3 Fig) are relatively small. This indicates that the differences between different regions are modest, and that the dynamics of pneumonia in all regions is mainly dominated by a seasonal cycle (~52 weeks periodicity in the wavelet spectra, see Fig 6). Although, the temporal patterns of pneumonia are very similar between the different regions at the small spatial scale of the Netherlands, they might be very different if we instead consider larger regions characterised by heterogeneous geographical characteristics (e.g. Europe). In countries such as Canada, previous studies have shown that there is a remarkable spatial variability in the temporal pattern of pneumonia [6]. Our results illustrate how wavelet analysis, which is not widely used yet in epidemiology (but see [12, 21] for some excellent examples), can be a useful tool to detect differences in temporal patterns when spatial spread [21] or geographical characteristics [22] play an important role.

The registration coverage of the hospital admissions has increased in 2014. It is therefore possible that the observed increase in pneumonia cases in 2014 reflects the change in the registration system. However, there was an early onset of the severe influenza epidemic of winter 2014/2015 and this could have resulted in hospital admissions in December 2014 [23]. More remarkable is the relatively high incidence of pneumonia hospital admissions in summer of 2014 (Fig 1). This suggests that atypical microorganisms such as *Legionella* species, *Mycoplasma pneumoniae* or *Chlamydia* species could have played a role (5). This clearly shows the need for more insight into microbiological causes of pneumonia.

Another aspect is that, to the best of our knowledge, no studies have been conducted to assess the sensitivity of the ICD-10 diagnoses. However, in a previous study, van den Garde and co-authors [19] assessed the sensitivity of the ICD-9 codes to be 79.5% for the principal and secondary codes. We therefore assume that this sensitivity would approximately be the same for the ICD-10 codes. In addition, one might argue that in our study community acquired pneumonia cannot be distinguished by hospital-acquired pneumonia. However, as pointed out in a previous study [24], the discharge diagnoses are representative of the reason for hospital admission and it is therefore unlikely that many cases have been incorrectly classified.

Information is also lacking on the causes of spatial variation in hospital admissions for pneumonia, that could include geographical differences in: 1. coding practices between hospitals; 2. general health status and health seeking behavior, associated with socio-economic status; 3. prevalence of underlying chronic diseases such as COPD; 4. referral practices of general practitioners; and 5. air pollution. Outdoor air pollution is known as a risk factor that increases the susceptibility to lower respiratory tract infections including (community-acquired) pneumonia [25, 26]. It might therefore be no coincidence that in our analysis the regions of high incidence detected are characterised by high population density (S2 Fig) or high farming activity (S2 Fig). In contrast, the regions with low incidence are characterised by low population density (S2 Fig) and low to medium farming activity (S2 Fig). One potential explanation is that people become more susceptible to pneumonia through exposure to substances emitted by livestock farms, traffic and industries, such as particulate matter, endotoxins and ammonia. A recent study in the intensively farmed south of the Netherlands shows that reduced lung function is strongly correlated with high concentration of ammonia, diffusing in the air from livestock farms. These effects are comparable with the harmful health effects caused by city traffic [4]. Regarding the effects of emissions from livestock farms, whereas the occurrence of respiratory effects is well established in farmers subject to exposure levels in stables [27, 28] there are also indications that lower ambient concentration levels affect the respiratory health of susceptible individuals living near livestock farms [29]. Amongst farm types in The Netherlands, poultry farms are producing relatively large emissions of particulate matter and endotoxins [30, 31]. In recent analyses of Dutch GP data it was found that living close to poultry farms was associated with an increased pneumonia risk [32–34].

## Conclusions

This study highlights that pneumonia hospital admissions are strongly clustered both in space and in time, and that even at a small spatial scale, differences in incidence can be substantial. It emphasizes that there is a need for more insight into the epidemiology of pneumonia, which is a prerequisite for devising prevention strategies. Although CAP is an important public health problem, remarkably little is known about its determinants. The identification of clear geographical clusters of high pneumonia incidence represents an important first step towards developing and testing hypotheses regarding these determinants.

## Supporting information

S1 Fig Maps of seasonal incidences of unspecified pneumonia cases at municipality levels for the years 2012, 2013 and 2014. Black circles represent significant clusters (p<0.05) identified whilst imposing a 10% upper limit and choosing a non-overlapping criterion.
(TIF)

S2 Fig Maps of population density and livestock density at municipality level in 2014. Population density per km^2^ (a), chickens density per km^2^ (b), pigs density per km^2^ (c) and goats density per km^2^ (d). The data on animal densities are from the Dutch Agricultural Census Register (Landbouwtelling Register, LBT). All the data in these maps are copyright of Statistics Netherlands, Den Haag/Heerlen.
(TIF)

S3 Fig Wavelet power spectra of the age adjusted incidences for the five regions in the Netherlands. In order to homogenize the variance the time series have first been square-root transformed and normalized. The time series have been clustered based on the similarity of their wavelet spectra. A cluster tree have been constructed (a) based on the dissimilarity matrix of the wavelets power spectra.
(TIF)

S4 Fig Maps of age adjusted incidence of unspecified pneumonia cases at municipality level for the years 2012, 2013 and 2014. In the main text, we considered a wide age group [0–25 years]. Because the etiology of pneumonia might differ between adults and children, we correct the incidence and the SatScan analysis using a less coarse classification, to check whether the classification of age classes would influence the main pattern. We divided the data in 6 age groups: [0–5); [5–15); [15–25); [25–45); [45–65) and 65+ (see also S1 Table). Black circles represent significant clusters (p<0.05) identified whilst imposing a 10% upper limit and choosing a non-overlapping criterion. The clusters in SaTScan have been adjusted by taking age classes as covariate in the analysis. The incidence intervals in the colorbar represent the quantiles of the pneumonia incidence in 2014. The spatial patterns obtained are identical to the one observed in Fig 3.
(TIF)

S5 Fig Maps of age adjusted incidence of unspecified pneumonia cases at municipality level for the years 2012, 2013 and 2014 for adults only. Because the etiology of pneumonia, might differ between adults and children, we removed from the dataset the infants [0–4] and the children [4–14]. Black circles represent significant clusters (p<0.05) identified whilst imposing a 10% upper limit and choosing a non-overlapping criterion. The clusters in SaTScan have been adjusted by taking age classes as covariate in the analysis. The incidence intervals in the colorbar represent the quantiles of the pneumonia incidence in 2014. The spatial patterns are in general similar to the patterns observed for the total population (Fig 3).
(TIF)

S6 Fig Maps of adjusted incidence of unspecified pneumonia cases at municipality level for the years 2012, 2013 and 2014. In the analysis we corrected for age, Socio Economic Status (SES) and for the level of urbanization (see S2 Table and S1 Appendix for details). Black circles represent significant clusters (p<0.05) identified whilst imposing a 10% upper limit and choosing a non-overlapping criterion. The clusters in SaTScan have been adjusted by taking age classes, SES and urbanization as covariate in the analysis. The incidence intervals in the colorbar represent the quantiles of the pneumonia incidence in 2014. The spatial patterns are in general similar to the patterns observed for pneumonia age corrected incidence only (Fig 3).
(TIF)

S1 Table Counts by age groups of hospital admissions for pneumonia in 2012, 2013 and 2014 for the ICD-10 discharge code J18, “pneumonia organism unspecified”. (DOCX)

S2 Table Class of Socio Economic Status (SES) and levels of urbanisation in the Netherlands. (DOCX)

S1 Appendix More detailed information on methods used. (DOCX)
