# Supplementary figures and images for: Space-time analysis of pneumonia hospitalisations in the Netherlands
Source: PLoS One. 2017 Jul 13;12(7):e0180797. doi: 10.1371/journal.pone.0180797 (PMC5509219; doi:10.1371/journal.pone.0180797)

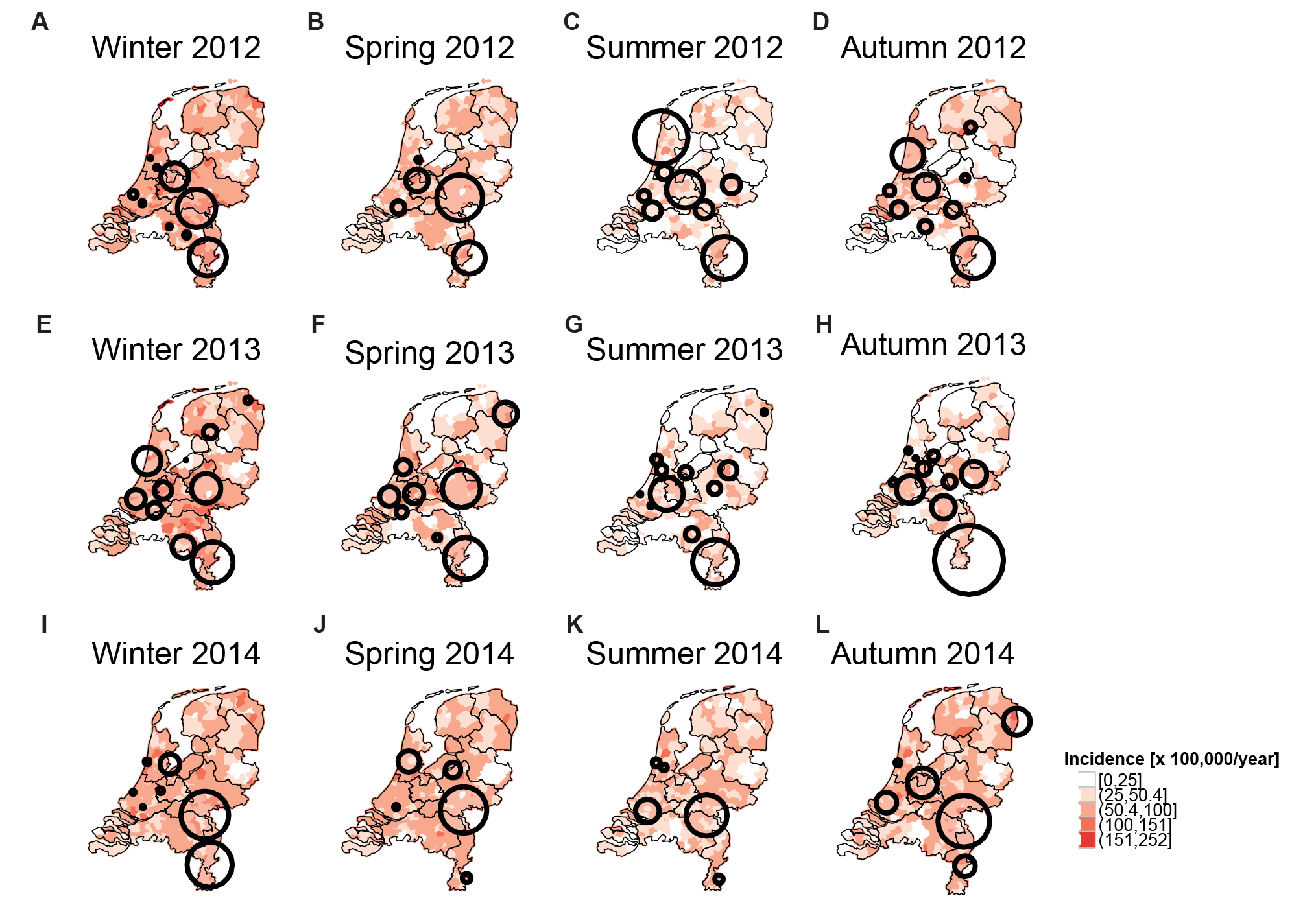

Supplement: S1 Fig — Black circles represent significant clusters (p<0.05) identified whilst imposing a 10% upper limit and choosing a non-overlapping criterion. (TIF) [file pone.0180797.s001.tif]

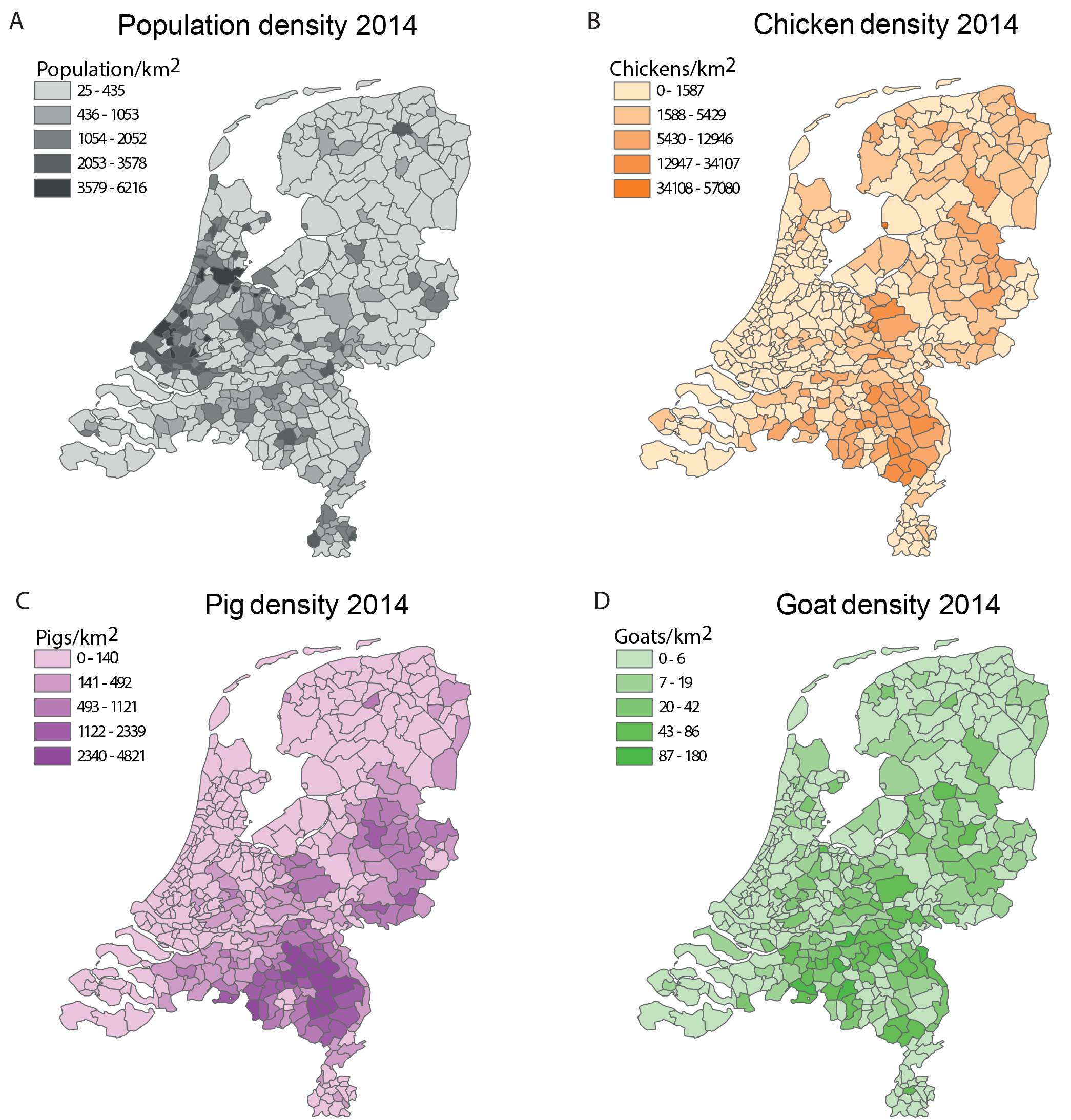

Supplement: S2 Fig — Population density per km2 (a), chickens density per km2 (b), pigs density per km2 (c) and goats density per km2 (d). The data on animal densities are from the Dutch Agricultural Census Register (Landbouwtelling Register, LBT). All the data in these maps are copyright of Statistics Netherlands, Den Haag/Heerlen. (TIF) [file pone.0180797.s002.tif]

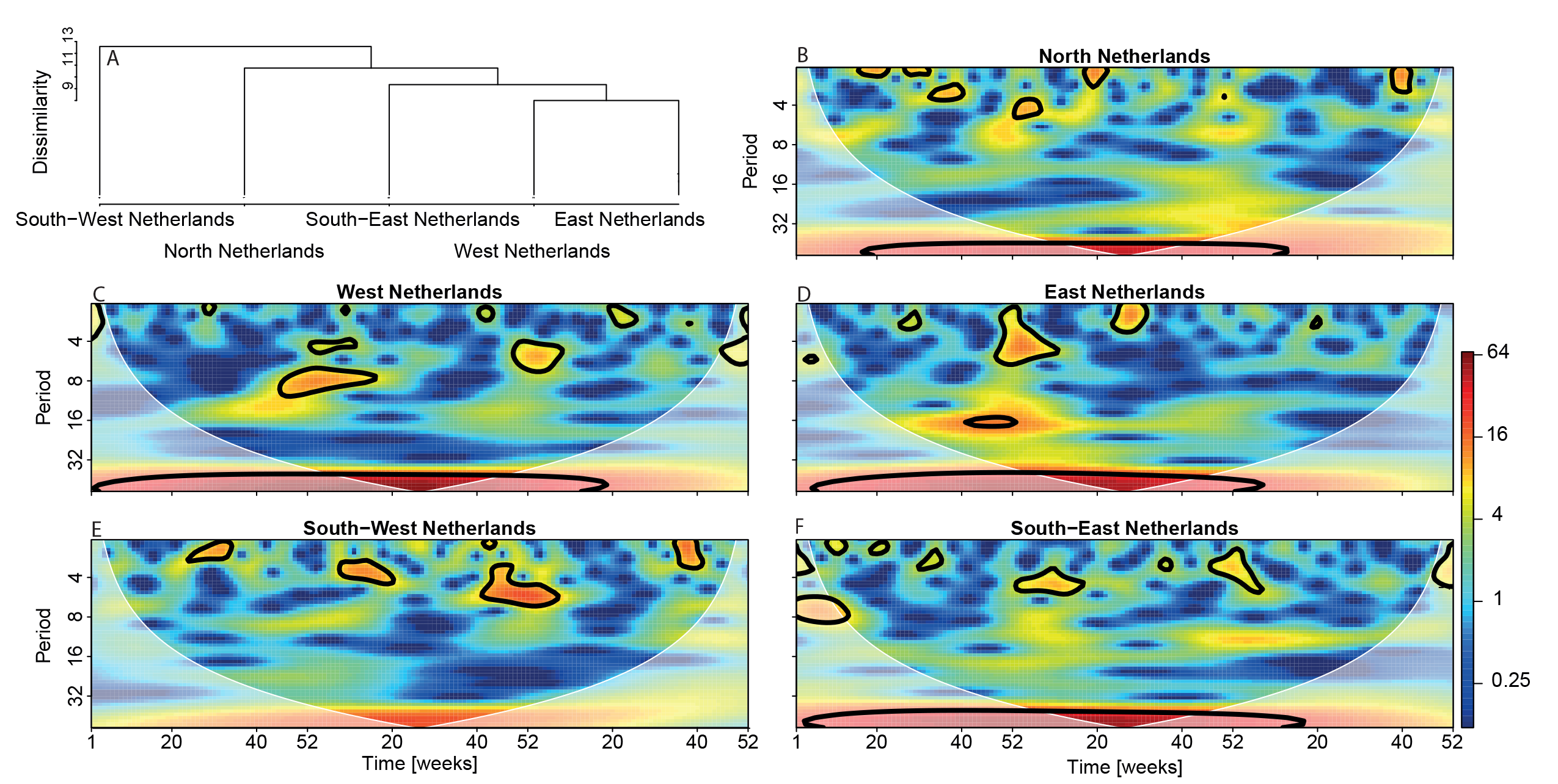

Supplement: S3 Fig — In order to homogenize the variance the time series have first been square-root transformed and normalized. The time series have been clustered based on the similarity of their wavelet spectra. A cluster tree have been constructed (a) based on the dissimilarity matrix of the wavelets power spectra. (TIF) [file pone.0180797.s003.tif]

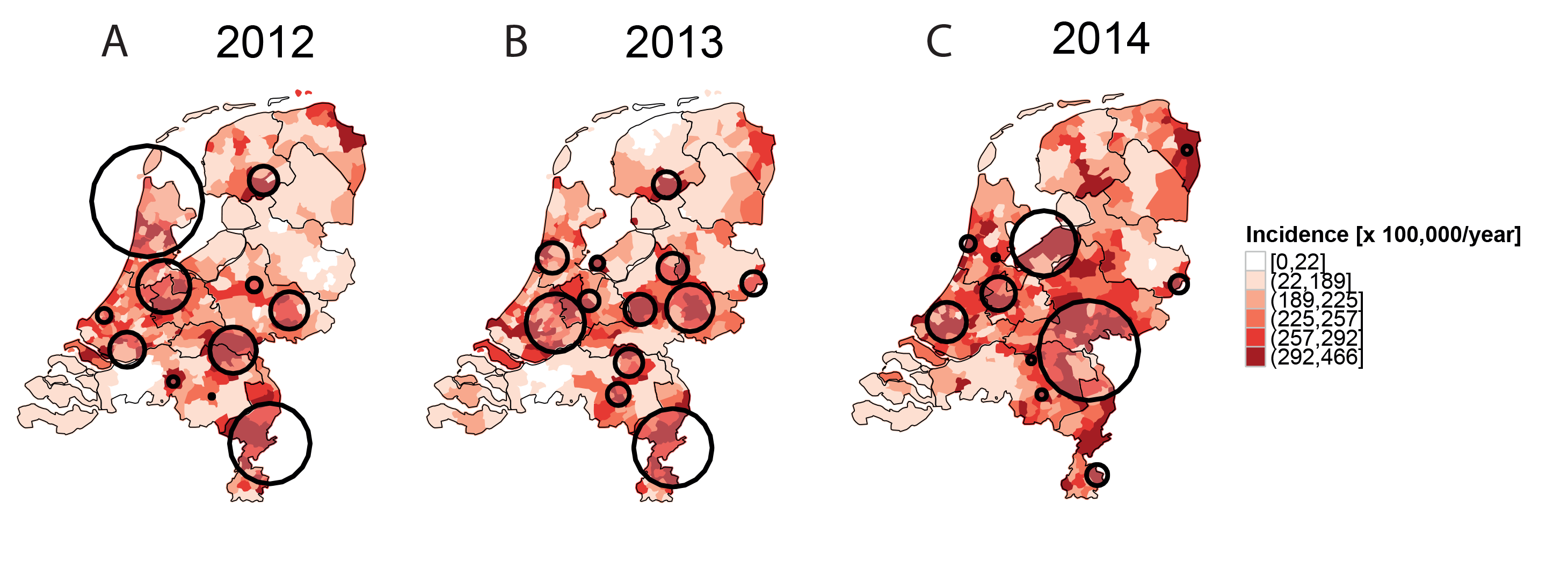

Supplement: S4 Fig — In the main text, we considered a wide age group [0–25 years]. Because the etiology of pneumonia might differ between adults and children, we correct the incidence and the SatScan analysis using a less coarse classification, to check whether the classification of age classes would influence the main pattern. We divided the data in 6 age groups: [0–5); [5–15); [15–25); [25–45); [45–65) and 65+ (see also S1 Table). Black circles represent significant clusters (p<0.05) identified whilst imposing a 10% upper limit and choosing a non-overlapping criterion. The clusters in SaTScan have been adjusted by taking age classes as covariate in the analysis. The incidence intervals in the colorbar represent the quantiles of the pneumonia incidence in 2014. The spatial patterns obtained are identical to the one observed in Fig 3. (TIF) [file pone.0180797.s004.tif]

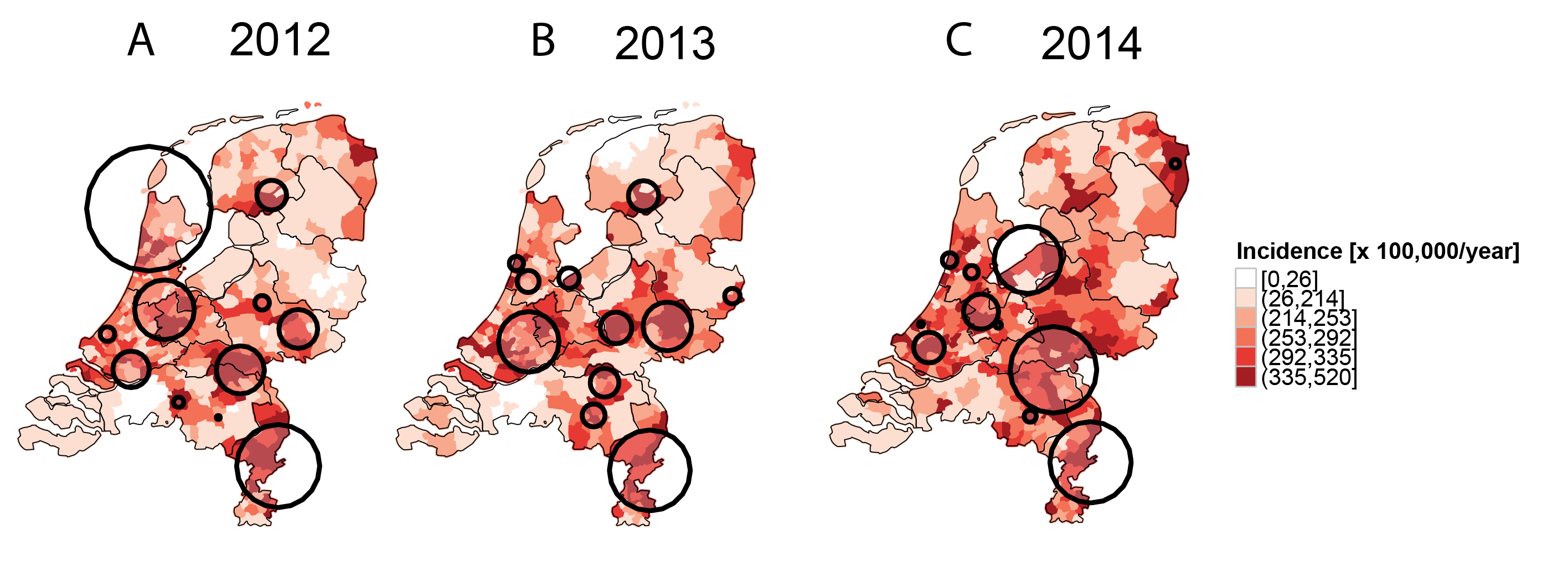

Supplement: S5 Fig — Because the etiology of pneumonia, might differ between adults and children, we removed from the dataset the infants [0–4] and the children [4–14]. Black circles represent significant clusters (p<0.05) identified whilst imposing a 10% upper limit and choosing a non-overlapping criterion. The clusters in SaTScan have been adjusted by taking age classes as covariate in the analysis. The incidence intervals in the colorbar represent the quantiles of the pneumonia incidence in 2014. The spatial patterns are in general similar to the patterns observed for the total population (Fig 3). (TIF) [file pone.0180797.s005.tif]

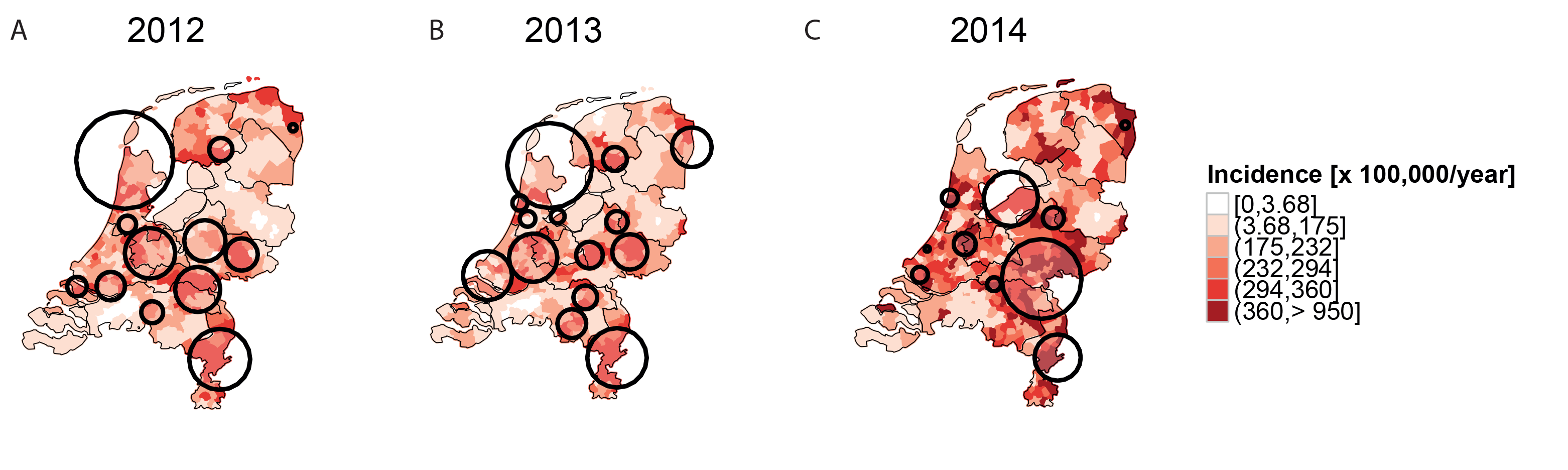

Supplement: S6 Fig — In the analysis we corrected for age, Socio Economic Status (SES) and for the level of urbanization (see S2 Table and S1 Appendix for details). Black circles represent significant clusters (p<0.05) identified whilst imposing a 10% upper limit and choosing a non-overlapping criterion. The clusters in SaTScan have been adjusted by taking age classes, SES and urbanization as covariate in the analysis. The incidence intervals in the colorbar represent the quantiles of the pneumonia incidence in 2014. The spatial patterns are in general similar to the patterns observed for pneumonia age corrected incidence only (Fig 3). (TIF) [file pone.0180797.s006.tif]
