# Supplementary material for: Space-time analysis of pneumonia hospitalisations in the Netherlands
Source: PLoS One. 2017 Jul 13;12(7):e0180797. doi: 10.1371/journal.pone.0180797 (PMC5509219; doi:10.1371/journal.pone.0180797)
Supplement: S1 Table — (DOCX) [file pone.0180797.s007.docx]

**S1 Table**

|  | **Age class** | | | | | |
| --- | --- | --- | --- | --- | --- | --- |
| **Year** | **[0-5)** | **[5-15)** | **[15-25)** | **[25-45)** | **[45-65)** | **≥65** |
| **2012** | 1778 | 679 | 450 | 2140 | 7118 | 24439 |
| **2013** | 1450 | 557 | 400 | 1966 | 7103 | 24651 |
| **2014** | 1670 | 770 | 526 | 2257 | 8506 | 28576 |
