# Supplementary material for: Space-time analysis of pneumonia hospitalisations in the Netherlands
Source: PLoS One. 2017 Jul 13;12(7):e0180797. doi: 10.1371/journal.pone.0180797 (PMC5509219; doi:10.1371/journal.pone.0180797)
Supplement: S2 Table — (DOCX) [file pone.0180797.s008.docx]

| SES score  minimum | SES score maximum | SES class | Description | Density addresses  [per km^2^] | Level of Urbanization | Description |
| --- | --- | --- | --- | --- | --- | --- |
| -3.41 | -1.50 | 1 | Very high | > 2500 | 1 | Highly urbanised |
| -1.5 | -0.5 | 2 | High | [1500-2500] | 2 | Urbanised |
| -0.5 | 0.5 | 3 | Medium | [1000-1500] | 3 | Moderately  Urbanised |
| 0.5 | 1.5 | 4 | Low | [500-1000] | 4 | Poorly urbanised |
| 1.5 | 5.24 | 5 | Very low | <500 | 5 | Not urbanised |

**S2 Table**
