# Supplementary material for: Space-time analysis of pneumonia hospitalisations in the Netherlands
Source: PLoS One. 2017 Jul 13;12(7):e0180797. doi: 10.1371/journal.pone.0180797 (PMC5509219; doi:10.1371/journal.pone.0180797)
Supplement: S1 Appendix — (DOCX) [file pone.0180797.s009.docx]

**S1 Appendix**

**Correction for age class, socio-economic-status and level of urbanisation**

Factors as socio economic status (SES) and level of urbanization might have an effect on pneumonia incidence, and we have therefore included these factors in the statistical analysis.

We use data on socio economic status and level of urbanisation in the Netherlands in 2010, made available by Statistics Netherlands, Den Haag/Heerlen. A SES score is available at each postal code. Based on the SES score, each postal code 4 level is assigned to a SES class (see S2 Table). The density of addresses per km^2^ determines the level of urbanization of each postal code (see S2 Table).

For each postal code 4 level we calculate the expected number of cases, by performing a Poisson regression to the observed number of cases (in R function glm, link function= Poisson). The log(population) as offset, and with explanatory factors the categorical variables, age class, SES and level of urbanisation. We used the 6 age classes defined in S1 Table. By knowing the expected number of cases and the observed number of cases one can calculate the standardised ratio, which is then used to correct the incidences. Consequently, we also re-performed the SatScan analysis by using the 6 age classes, the Socio Economic Status and the level of urbanisation. The so corrected incidences and the corresponding SatScan analysis are shown in S6 Fig.

**Wavelet analysis**

The description of wavelet analysis presented here follows the supplementary information provided in [1] and the interested reader is referred to it for further details. Wavelet analysis makes use of a wavelet function, which is a periodic function resembling a local wave. By definition, wavelet functions have zero mean and are localized in frequency and time. One particular wavelet function, called the Morlet wavelet, consists of the product of a sine wave and a Gaussian bell-shaped curve:

$\Psi_{0}\left( \eta\right)=\pi^{-1/4}e^{i \omega_{0}\eta}e^{{-\eta}^{2}/2}$ (1)

where *i*=$\sqrt{-1}$, ω_0_ is dimensionless frequency, and η is dimensionless time. The Morlet wavelet

is particularly useful to analyze periodicities in time series. Therefore, we chose the Morlet

wavelet for our analysis, assuming the standard value of ω_0_=6 to satisfy the admissibility

condition for wavelet functions [2].

We consider a time series *x_n_*, consisting of observations *n*=1,…,*N* that are equally spaced

in time at intervals *δt.* The continuous wavelet transform, *W_n_* (s), of our discrete time series *x_n_* is

defined as the convolution of *x_n_* with the scaled and normalized wavelet. This can be written as [3]:

$W_{n}\left( s \right)=\surd\frac{\delta t}{s}\sum_{m=1}^{N} x_{m}\Psi_{0}\left[ \frac{(m-n)\delta t}{s} \right]$ (2)

The wavelet transform is stretched in time by varying the wavelet scale *s*. The scales *s* are usually chosen as *s_j_=s_0_2^jδs^*_,_ with *j=0,1,..,J.* where *_S0_* is the smallest resolvable scale *and J* provides the maximum scale*.* We used the following values: *δt=1, δs=1/12, s_0_=2δt,* and *J=57*. The wavelet transform is normalized to ensure that wavelet transforms at different scales s are comparable. The local wavelet power spectrum is defined as *W_n_(s) ^2^*, whereas the complex argument of *W_n_(s)* can be interpreted as the local phase [3, 4]. The word ‘local’ indicates that the wavelet power spectrum and its phase depend on the local time *n*. Then, the local wavelet power spectrum is plotted in contour plots as a function of time (see Fig6b-f). Color codes represent wavelet power, which measures how much variance of the time series is explained by each periodicity. Areas inside the black contour lines correspond to 95% confidence regions where the power is higher than the power of red noise with the same autocorrelation coefficient as the data. Transparent areas on the left and right hand sides of the plots represent the cone of influence, which is a region where edge effects are important. More detailed information on wavelet analysis can be found in [1, 3-7].

**Wavelet cluster analysis**

Once computed the wavelet spectrum of each of the time series we need to quantify the similarity between the patterns. For this purpose, a Maximum Covariance Analysis (MCA) has been applied [8, 9].

First, the method calculates a covariance matrix *M_ij_* between each couple of wavelet spectra W_i_ and W_j_ :

$M_{i,j}$=$W_{i}W_{j}^{t}$, (8),

where the superscript *t* indicates transposition.

Subsequently, a singular value decomposition is performed on *M_i,j_* as follow:

${M_{i,j}=U\Gamma V}^{t}$ (9),

where *U* and *V* are unitary matrices and their columns, which are respectively the singular vectors for *W_i_* and *W_j_*  which obey an orthogonality relation. The elements of the diagonal matrix Γ (i.e. the singular values) are proportional to the squared covariance explained by each axis and they are ordered from the highest to the lowest.

From here, one can project the spectra *W_i_* and *W_j_* onto their respective singular vectors and calculate a the k^th^ so-called leading patterns:

$L_{i}^{k}\left( t \right)=\sum_{f=1}^{f=F} U^{k}\times W_{i}(f,t)$ (10)

$L_{j}^{k}\left( t \right)=\sum_{f=1}^{f=F} U^{k}\times W_{j}(f,t)$ (11),

with *F* being the maximum frequency common to both spectra.

Then, the distance between the two spectra is computed as follow:

$D\left( i,j \right)=\frac{\sum_{k=1}^{k=K} c_{k}\times(D\left( L_{i}^{k},L_{j}^{k} \right)+D(U_{i}^{k},V_{j}^{k}))}{{\sum_{k=1}^{k=K} c}_{k}}$,

being *c_k_* the amount of covariance explained by each axis.

A dissimilarity matrix is then calculated, where each element is obtained by applying eq. 11 in turn to each pair of wavelet spectra. Then, the dissimilarity matrix is used to construct a cluster tree (see Fig 6a). Further information on wavelet clustering and its application in ecological and epidemiological studies can be found in [8-10] .

**References**

1. Benincà E, Ballantine B, Ellner SP, Huisman J. Species fluctuations sustained by a cyclic succession at the edge of chaos. Proceedings of the National Academy of Sciences of the United States of America. 2015;112(20):6389-94. Epub 2015/04/23. doi: 10.1073/pnas.1421968112. PubMed PMID: 25902520; PubMed Central PMCID: PMCPmc4443354.

2. Farge M. Wavelet transforms and their applications to turbulence. Annual review of fluid mechanics. 1992;24(1):395-458.

3. Grinsted A, Moore JC, Jevrejeva S. Application of the cross wavelet transform and wavelet coherence to geophysical time series. Nonlinear processes in geophysics. 2004;11(5/6):561-6.

4. Torrence C, Compo GP. A practical guide to wavelet analysis. Bulletin of the American Meteorological society. 1998;79(1):61-78.

5. Cazelles B, Chavez M, Magny GC, Guegan JF, Hales S. Time-dependent spectral analysis of epidemiological time-series with wavelets. Journal of the Royal Society, Interface / the Royal Society. 2007;4(15):625-36. Epub 2007/02/16. doi: 10.1098/rsif.2007.0212. PubMed PMID: 17301013; PubMed Central PMCID: PMCPmc2373388.

6. Cazelles B, Chavez M, Berteaux D, Ménard F, Vik JO, Jenouvrier S, et al. Wavelet analysis of ecological time series. Oecologia. 2008;156(2):287-304.

7. Benincà E, Jöhnk KD, Heerkloss R, Huisman J. Coupled predator–prey oscillations in a chaotic food web. Ecology letters. 2009;12(12):1367-78.

8. Rouyer T, Fromentin JM, Stenseth NC, Cazelles B. Analysing multiple time series and extending significance testing in wavelet analysis. Marine Ecology Progress Series. 2008;359:11–23.

9. Rouyer T, Fromentin J-M, Ménard F, Cazelles B, Briand K, Pianet R, et al. Complex interplays among population dynamics, environmental forcing, and exploitation in fisheries. Proceedings of the National Academy of Sciences. 2008;105(14):5420-5.

10. Ben-Ari T, Neerinckx S, Agier L, Cazelles B, Xu L, Zhang Z, et al. Identification of Chinese plague foci from long-term epidemiological data. Proceedings of the National Academy of Sciences of the United States of America. 2012;109(21):8196-201. Epub 2012/05/10. doi: 10.1073/pnas.1110585109. PubMed PMID: 22570501; PubMed Central PMCID: PMCPmc3361404.
